# Zebrafish *aplnra *functions in epiboly

**DOI:** 10.1186/1756-0500-2-231

**Published:** 2009-11-19

**Authors:** Svanhild Nornes, Ben Tucker, Michael Lardelli

**Affiliations:** 1 Centre for the Molecular Genetics of Development and Discipline of Genetics, School of Molecular and Biomedical Science, The University of Adelaide, 5005, S.A., Australia

## Abstract

**Background:**

The zebrafish, *Danio rerio*, possesses the paralogous genes *aplnra *and *aplnrb *that are duplicates of an ancestral orthologue of the human *APLNR *gene encoding a G-protein coupled receptor that binds the peptide ligand APELIN and is required for normal cardiovascular function. *aplnrb *is required for migration of cells contributing to heart development in zebrafish embryos. *aplnra *is transcribed in a complex pattern during early development but its function in embryogenesis is largely unknown.

**Findings:**

Blockage of translation of *aplnra *mRNA in zebrafish embryos results in retarded or failed epiboly with the blastoderm apparently disconnected from the nuclei of the yolk syncytial layer. Gastrulation is also defective. Failure of correct tail extension is observed with ectopic structures resembling somites positioned dorsal to the spinal cord.

**Conclusion:**

*aplnra*, unlike its duplicate *aplnrb*, is essential for normal epiboly, although this function appears to be independent of signalling activated by zebrafish Apelin. The defects in epiboly caused by loss of *aplnra *activity appear, at least partially, to be due to a requirement for *aplnra *activity in the yolk syncytial layer.

## Background

The human gene *APLNR *(formerly named *AGTRL1*) encodes a G protein-coupled receptor related to the angiotensin receptors. Investigation in a variety of model systems has shown that APLNR has roles in both development of the blood vasculature and the heart [1-5] and in regulation of cardiovascular function, e.g. as a potent stimulator of cardiac contractility [6]. It also functions as an arterial and venous dilator [7]. APLNR has also been of interest as a receptor for the Human Immunodeficiency Virus, [8].

The zebrafish genes *aplnra *(formerly *agtrl1a*) and *aplnrb *(formerly *agtrl1b*) are paralogous genes derived by duplication of an ancestral teleost orthologue of human *APLNR*. *aplnrb *has been shown to be essential for the earliest moments of heart morphogensis (the formation of myocardial progenitors in the heart field and their subsequent migration) and for cell movements during gastrulation [4,5]. These two genes appear to have somewhat redundant functions in formation of cardiomyocytes [4]. *aplnra *and *aplnrb *are transcribed in similar patterns during epiboly and gastrulation suggesting that they may also both play roles in controlling these processes [4,5,9]. While loss of Aplnrb protein expression causes defects in gastrulation, no role in epiboly has been observed.

The ligand for human APLNR protein is a short peptide, APELIN (reviewed by [10]), for which a zebrafish orthologue exists encoded by the gene *apln*. Zeng et al. [5] detected *apln *transcripts (by RT-PCR) first during development after commencement of gastrulation. By mid-gastrulation, *apln *transcripts can be detected in the notochord by whole mount *in situ *transcript hybridisation. This argues against a role for zebrafish Apelin-Aplnra/b signalling in epiboly. However, ectopic and early expression of *apln *mRNA by mRNA injection does produce defects in epiboly [5] suggesting that inappropriate activation of Aplnra/b receptors might interfere with this morphological movement.

## Materials and methods

The work was carried out under the auspices of the Animal Ethics and Institutional Biosafety Committees of the University of Adelaide.

Morpholino (MO) oligonucleotides were obtained from Gene Tools (LLC, Corvallis, OR, USA). Three *aplnra*-MOs binding to non-overlapping sequences on *aplnra *mRNA to block translation were designed as follows:

(MO1) 5'-GTGTATTCCGACGTTGGCTCCATTT-3'

(MO2) 5'-TTGAGTCCTTCTTGAGCAGTTTATC-3'

(MO3) 5'-AATGTTTGGATTCTTCTGTCTGATA-3'.

The standard negative control MO sequence (Cont MO), also from Gene Tools, had the sequence: 5'-CCTCTTACCTCAGTTACAATTTATA-3'. Rescue of the phenotype generated by MO3 injection was demonstrated by injection of this MO at the 1-cell stage followed by injection of *aplnra *mRNA (see later) that had been engineered to lack the MO3-binding site and to contain an optimal Kozak's sequence (5'-GCCAGG**ATG**-3', bold indicates start codon). This mRNA was transcribed from the pCS2+ expression vector containing the *aplnra *open reading frame using the mMessage mMachine kit (Ambion Inc., Austin, TX, USA). 2-5 nL of morpholino or mRNA were injected per embryo. MO and mRNA injections, [11] and whole mount *in situ *transcript hybridisation, [12], were performed essentially as previously described.

To visualise migration of YSL nuclei relative to the blastoderm margin during epiboly, embryos injected with Cont MO or MO3 were subsequently injected with 1 nL of 0.5 mM Sytox Green fluorescent nucleic acid dye (Molecular Probes^®^, Invitrogen, Life Technologies Corp. Carlsbad, Ca, USA) into the yolk cell at the sphere stage (4 hours post fertilisation @ 28.5°C, hpf) and then visualised at 6-8 hpf under both epifluorescence and bright field optics using a Zeiss AxioImager Z.1 with ApoTome attachment. Images were captured and processed using a Zeiss AxioCam MR and AxioVision 4.5 software (Carl Zeiss MicroImaging GmbH, Jena, Germany).

## Results and Discussion

### Aplnra is required for normal epiboly

**Figure 1:**
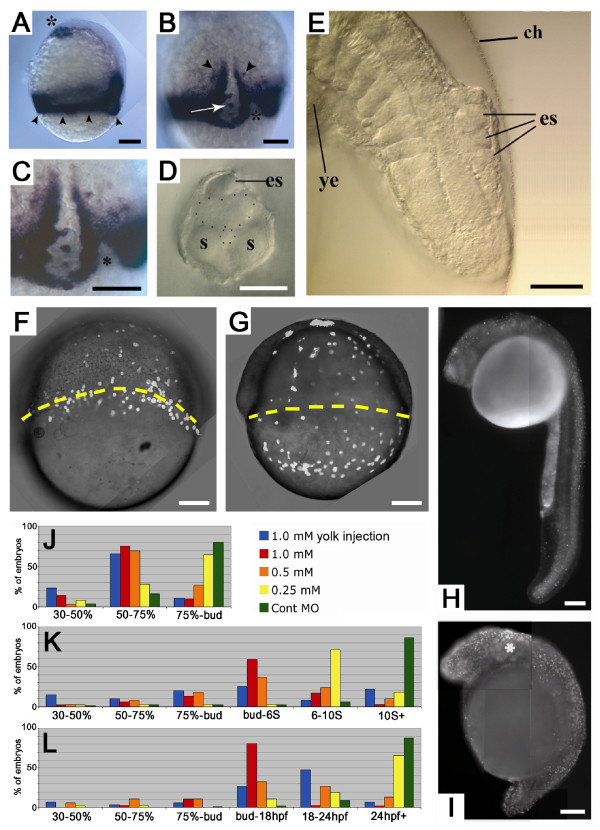
**Phenotypic effects of blocking translation of *aplnra *mRNA**. **A-C**: Lateral (**A**) and dorsal axial views (**B, C**) of a representative embryo at 75% epiboly injected with MO oligonucleotide inhibiting translation of *aplnra *mRNA. Anterior is up. This embryo is stained to show cells transcribing *tbx16 *in order to reveal paraxial presomitic mesoderm. The position of the arrested epibolic margin is indicated by arrowheads in **A**. Note the presence of *tbx16*-expressing cells in the axis (white arrows in **B**, enlarged in **C**), and apparently scattered in the hypoblast (arrowheads in **B**), and notches in the margin (asterices in **B, C**). **D-E**: Ectopic structures resembling somites (es) in the retarded tails of embryos with reduced *aplnra *mRNA translation but that complete epiboly. These ectopic structures are clearly seen in a lateral view (**E**, anterior to top, dorsal to right). The yolk extension (ye) and chorion (ch) are indicated. A transverse optical section of the tail tip shows ectopic structures resembling somites (es) dorsal to the spinal cord (**D**). **F-G**: YSL nuclei, labelled with Sytox Green dye (white dots) move vegetally with the blastoderm margin in an embryo injected with Cont MO at 1 mM (**F**) but move ahead of the margin in an embryo injected with MO3 at 1 mM (**G**). Yellow dashed lines indicate the position of the blastoderm margin. Embryos are oriented with animal pole to top. Each image is a composite of images captured under epifluorescence and bright field illumination. **H**-**I**: Acridine orange staining of a Cont MO-injected embryo (**H**) and an embryo injected with MO3 at 1 mM at the 1-cell stage (**I**). The latter embryo shows a slightly higher level of apoptosis, primarily in the head (asterisk). Note the retarded tail formation. Anterior is left and dorsal is up. **J-L**: Embryos were injected with Cont MO at 1 mM or with a series of MO2 concentrations (1 mM, 0.5 mM, 0.25 mM) blocking *aplnra *mRNA translation at the one cell stage or into the animal pole of the yolk cell at the 32-cell stage (1 mM yolk injection). The injected embryos were observed at 8 (**a**), 14 (**b**) and 24 hpf (**c**) and assessed for the extent to which they had undergone epiboly and, if they completed epiboly, for the length of their tail extension. Developmental stage descriptors under the histograms refer only to epiboly or tail extension relative to that of a wild type embryo at 28.5°C. Size bars are 100 μm except for D, E, 50 μm.

To test the effects on development of loss of Aplnra protein activity we designed three antisense MO oligonucleotides (MO1, MO2, MO3) complementary to non-overlapping regions of the 5' untranslated region or translational start region of *aplnra *mRNA. Injection of each of these MOs into 1 cell-stage embryos resulted in complete or partial failure of epiboly in a concentration dependent manner (Table 1; Figure 1J). In these embryos, cleavage and blastula stages appeared normal, but by the onset of gastrulation, a slight retardation of epiboly was evident. This delay subsequently increased relative to negative control MO (Cont MO)-injected embryos such that when control embryos had completed epiboly (bud stage, Figure 1J), the vegetal progress of the gastrula margin of *aplnra*-MO injected embryos was frequently stalled prior to yolk plug closure at the 75% position (Figure 1J and arrowheads in Figure 1A). Nevertheless, at this stage, the head process had migrated to an essentially wildtype position at the animal pole (asterisk, Figure 1A). Many of the stalled embryos subsequently burst their vegetal yolk cells, leading to death (data not shown). By the time that control siblings reached the 10 somite stage (Figure 1K), *aplnra *MO-injected embryos that had completed epiboly showed a concentration-dependent delay in tailbud formation and posterior extension, and concentration-dependent severity of defects in these processes. This led to a failure in tail and yolk extension eversion and outgrowth, when assayed at 24 hpf (Figure 1L, and compare H and I).

**Table 1:** *aplnra *function is required for normal epiboly and elongation/extension movements

		%				
		
Age (normal developmental stage)	Apparent Developmental Stage^a^	A	B	C	D	Control
**8 hpf (75% epiboly)**	75% epiboly-bud	11	10	27	65	80
	50%-75% epiboly	66	75	70	28	16
	30%-50% epiboly	23	14	3	8	4
	
	(n) =	(96)	(69)	(60)	(65)	(48)
						
**14 hpf (10 somite)**	10 somite stage+	22	3	10	18	87
	6-10 somite stage	8	17	24	71	6
	bud-6 somite stage	25	59	37	3	2
	75% epiboly-bud	20	13	18	3	2
	50% - 75% epiboly	10	6	8	3	2
	30%-50% epiboly	15	2	3	2	1
	
	(n) =	(61)	(57)	(62)	(65)	(46)
						
**24 hpf (prim-5)**	24 hpf+	7	2	13	66	88
	18-24 hpf	48	3	27	19	9
	bud-18 hpf	27	81	33	11	2
	75% epiboly-bud	6	11	11	0	1
	50% - 75% epiboly	4	3	11	3	0
	30%-50% epiboly	7	0	6	2	0
	
	(n) =	(67)	(63)	(64)	(64)	(45)
						
	
	Ectopic Cell Mass^b^	67	84	17	4	1

Injection of MO2 at different concentrations results in different levels of developmental delay in terms of epiboly and elongation/extension. Injection of MO2 at later developmental stages (into yolk cell vegetal to embryo at 32-cell stage, partially restricting localisation of MO2 to the YSL) leads to similar, if slightly reduced, levels of delay as found in embryos injected at the 1-cell stage. This indicates that *aplnra *has critical functions primarily in the YSL. A = 1 mM MO2 injection at 32 cell stage (animal pole of the yolk), B = 1 mM MO2 injection at 1 cell stage, C = 0.50 mM MO2 injection at 1 cell stage, D = 0.25 mM MO2 injection at 1 cell stage, Control = 1 mM MOCont injection at 1 cell stage. Notes: ^a ^As determined by extent of epiboly or extension/elongation. ^b ^Ectopic cell mass located dorsally to the early tail-bud/origin of the yolk extension, depending on apparent developmental stage. Represented as % of the total number of embryos injected (24 hpf).

In contrast to the striking defective epiboly phenotypes seen with the *aplnra*-targeted MOs, injection of Cont MO at the highest of these concentrations (1.0 mM) produced a very low level of defects (Figure 1J-L, Table 1). Furthermore, injection of MO3 at the 1-cell stage followed by injection into the yolk cell of an *aplnra *mRNA engineered to lack MO3's binding site led to effective rescue of the epiboly phenotype (described later). We also examined whether the failure to undergo posterior extension might be caused by cell death in the tailbud. Acridine orange staining of *aplnra*-injected embryos revealed a slight increase in the number of dying cells with elevated levels found primarily in the head (asterisk, Figure 1I) relative to Cont MO-injected siblings (Figure 1H). Combined, these data support that the defective epiboly phenotype is a specific effect of reducing the translation of endogenous *aplnra *mRNA, indicating a role for this gene in the regulation of epiboly.

*aplnra *is expressed in the yolk syncytial layer and the enveloping layer (EVL) [9]. Microtubules within the YSL are required to drive normal epibolic movement and this is achieved through correct connections with the EVL. The published data on *aplnrb *expression are insufficient to determine whether this gene is also expressed in the YSL and EVL. During early epiboly, *aplnra *is also expressed in deep cells [9]. At the 32-cell stage, central blastomeres no longer have cytoplasmic connections to the yolk cell [13]. If a MO was injected into the yolk at the 32-cell stage, its distribution would be restricted mainly to the YSL and marginal blastomeres and, at later stages, many cells of the embryo proper would possess no or a lower concentration of the MO. When MO2 was injected into the yolk cell at the 32-cell stage, the embryos still showed defects in epiboly but, in those embryos that managed to complete epiboly, there was a decrease in tail extension defects. This supports that the tail extension defects are primarily due to lack of *aplnra *function in the embryo proper (Table 1).

We were unable to rescue epiboly defects in MO3-injected embryos when both MO3 and *aplnra *mRNA (engineered to lack the MO3-binding site) were injected into the embryo at the one cell stage (data not shown). However, we observed a highly statistically significant decrease in epiboly defects when MO3 was injected at the 1-cell stage followed by *aplnra *mRNA injection into the yolk cell immediately vegetal to the embryo at the 32-cell stage (Table 2). The failure of mRNA injection to rescue development when this was performed at the 1-cell stage but success after injection at the 32-cell stage may reflect instability of injected mRNA and/or its protein product resulting in failure to provide sufficiently high concentrations of *aplnra *activity at the required developmental stage. Another possibility is that injection of mRNA into the yolk cell immediately vegetal to the embryo at the 32-cell stage provides greater *aplnra *activity in the YSL compared to when mRNA is injected into the embryo proper at the 1-cell stage. This is because mRNA injected into zebrafish embryos has a much lesser tendency to spread than MO oligonucleotides as demonstrated by the frequently unilateral function of mRNA when injected into one cell of a 2-cell stage embryo (e.g. [14]). The result above supports that *aplnra *activity in the YSL is important for normal epiboly in zebrafish.

**Table 2:** Rescue of the *aplnra *MO epiboly phenotype by co-injection of *aplnra *mRNA

Extent of epiboly at 8 hpf	MO3 alone	MO3 + mRNA
	% (n)	% (n)
**75% epiboly - bud**	4.8 (7)	21 (30)

**50-75% epiboly**	49.0 (71)	52.4 (75)

**30-50% epiboly**	46.2 (67)	26.6 (38)

**Total number injected (n)**	(145)	(143)

MO3 at 1 mM was injected into the embryos at the 1-cell stage. *aplnra *mRNA at 100 ng/μL was injected into the yolk cell immediately vegetal to the embryo at the 32-cell stage. A contingency χ^2 ^test of the data across all the phenotypic classes gives p < 0.001.

Loss of *aplnra *function by injection of MO3 results in decoupling of YSL nuclei from the blastoderm margin as demonstrated by labelling of YSL nuclei with Sytox Green dye (Figure 1F, G). Note that we are unable to state whether this uncoupling is due to loss of *aplnra *function in the YSL, the EVL or both since we have not examined the behaviour of the EVL when *aplnra *activity is lost.

### Aplnra is required for normal cell movement during gastrulation

Close examination of the gastrula margin and presumptive notochord in *aplnra *MO-injected embryos using the transcription of *tbx16 *to highlight these structures revealed several types of disruption (Figure 1A-C). Firstly, the margin was often uneven near the shield, with groups of cells apparently displaced animally from their neighbours (asterices, Figure 1B, C). These "notches" in the margin have previously been seen in *cdh1 *(*hab*) mutants [15], suggesting a problem with cell adhesion. Secondly, isolated, scattered *tbx16 *positive cells were seen in the hypoblast animal to the marginal expression domain (arrowheads, Figure 1B). Thirdly, although the shield and notochord/spinal cord primordia are normally devoid of *tbx16 *expressing cells, in *aplnra*-deficient embryos multiple *tbx16 *positive cells were found in this domain (arrows, Figure 1B, C). Zeng et al. [5] showed that *apelin *is expressed in the notochord during gastrulation and both *aplnra *[9] and *aplnrb *[5] are expressed in the epithelial adaxial cells that flank this structure. Thus, the loss of *aplnra *expression appears to allow either mixing of axial and adaxial cells or inappropriate differentiation of cells within axial structures. Furthermore, in many of the embryos surviving to tail extension stages, structures resembling somites were observed lying dorsal to the CNS (Figure 1D, E). These observations of aberrant positioning of cells of possible paraxial origin strongly suggest that multiple cell movements are affected by reduction of *aplnra *function.

## List of Abbreviations Used

*aplnra*/*b*: zebrafish *Apelin receptor a *or *b *gene; Aplnra/b: zebrafish Apelin receptor a or b protein; EVL: enveloping layer; hpf: hours post fertilisation @ 28.5°C; MO: morpholino; YSL: yolk syncytial layer.

## Competing interests

The authors declare that they have no competing interests.

## Authors' contributions

Both SN and BT contributed equally to analysis of the phenotypic effects of blocking Aplnra translation by MO injection. SN and BT were also both involved in drafting of the manuscript. ML designed the research project, performed some embryo photography and contributed to drafting the manuscript. All authors read and approved the final manuscript.
